# Eribulin mesylate in combination with ivonescimab as first-line treatment for unresectable locally advanced or metastatic triple-negative breast cancer(EMIT): study protocol for a single-arm, multicenter clinical trial

**DOI:** 10.3389/fimmu.2026.1927990

**Published:** 2026-10-21

**Authors:** Haiqi Lu, Xufei Wang, Zhuo Wang, Miaoqin Chen, Yiming Zhong, Lingfeng Ma, Wanfen Tang, Xian Wang, Guangliang Li

**Affiliations:** 1 Department of Medical Oncology, Sir Run Run Shaw Hospital, Zhejiang University School of Medicine, Hangzhou, Zhejiang, China; 2 Nursing Department, Sir Run Run Shaw Hospital, Zhejiang University School of Medicine, Hangzhou, Zhejiang, China; 3 Laboratory of Cancer Biology, Key Lab of Biotherapy in Zhejiang Province, Cancer Center of Zhejiang University, Sir Run Run Shaw Hospital, Zhejiang University School of Medicine, Hangzhou, Zhejiang, China; 4 Department of Medical Oncology, Jinhua Central Hospital/Affiliated Jinhua Hospital, Zhejiang University School of Medicine, Jinhua, Zhejiang, China; 5 Department of Breast Medical Oncology, Zhejiang Cancer Hospital, Hangzhou Institute of Medicine (HIM), Chinese Academy of Sciences, Hangzhou, Zhejiang, China

**Keywords:** Eribulin Mesylate, immunotherapy combined with anti-angiogenesis, ivonescimab, PD-L1, study protocol, triple negative breast cancer

## Abstract

**Background:**

Advanced triple-negative breast cancer (TNBC) has an extremely poor prognosis, and current therapies offer limited benefits for patients with low programmed cell death ligand 1(PD-L1) expression. This study innovatively proposes a “novel chemotherapy + bispecific antibody” model. Eribulin Mesylate (hereinafter referred to as Eribulin), a novel microtubule inhibitor, kills tumor cells while remodeling the tumor microenvironment. When combined with the PD-1/VEGF bispecific antibody Ivonescimab, it concurrently dismantles immunosuppression and angiogenesis to synergistically overcome immunotherapy resistance.

**Methods:**

This is a multicenter, open-label, single-arm clinical trial designed to enroll 29 patients with locally advanced or metastatic TNBC who have not received prior systemic anticancer therapy, and who are either ineligible for radical surgical resection or local therapy, or have experienced disease progression following postoperative adjuvant therapy. The primary endpoint is the objective response rate (ORR) assessed by Investigator Assessment (INV) at each participating center based on strict adherence to Response Evaluation Criteria in Solid Tumors version 1.1(RECIST v1.1) criteria. Secondary endpoints include progression-free survival (PFS), overall survival (OS), duration of response (DoR), and safety. Exploratory endpoints involve potential biomarkers, the correlation between PD-L1 expression and efficacy, and patient-reported outcomes (PROs).

**Discussion:**

This combination regimen is expected to leverage the synergistic advantages of “chemotherapy-promoted immunity and potent bispecific antibody efficacy,” providing novel evidence-based medical evidence for the first-line treatment of metastatic TNBC and thereby effectively addressing significant unmet clinical needs.

**Clinical Trial Registration:**

http://www.chictr.org.cn/, identifier ChiCTR2600117652.

## Background

As the most common malignant tumor among women worldwide, breast cancer has become the “First Killer” severely threatening women’s health (2). Among all breast cancer subtypes (i.e., Luminal A, Luminal B, HER2-positive, and TNBC), TNBC accounts for approximately 15%-20% (3). Due to the lack of estrogen receptor (ER), progesterone receptor (PR), and human epidermal growth factor receptor 2 (HER2) expression, TNBC is insensitive to endocrine and targeted therapies (4). It also exhibits high invasiveness and a tendency for early relapse and metastasis. Currently, it is the subtype with the poorest clinical efficacy and prognosis among all breast cancers, often referred to as “the most malignant breast cancer,” with a 5-year survival rate of only 12% for advanced-stage patients (5, 6). Although emerging agents such as immune checkpoint inhibitors and antibody-drug conjugates have shown great promise in TNBC treatment in recent years, the currently proven effective systemic treatment remains chemotherapy based on anthracyclines and/or taxanes. However, patients often experience relapse or distant metastasis shortly after completing chemotherapy, leading to poor survival outcomes. Furthermore, the severe toxic side effects of multi-line chemotherapy significantly impair patients’ quality of life and limit long-term treatment benefits. Therefore, exploring effective targeted therapeutics and molecular targets for combination with chemotherapy to improve efficacy has become a critical clinical challenge requiring urgent attention (7).

Previous studies have confirmed high levels of tumor-infiltrating lymphocytes (TILs) and PD-L1 protein expression in TNBC, indicating active immune responses (8, 9). Scoring via TILs counting or immune markers has revealed that TNBC patients with high TILs expression exhibit better prognoses, even among those who have not received adjuvant or neoadjuvant chemotherapy (10). These results provide a strong theoretical basis for the use of immunotherapy in TNBC. In addition, literature has reported that treating human or murine TNBC cells with carboplatin, doxorubicin, gemcitabine, or paclitaxel can induce coordinated transcriptional induction of PD-L1 mRNA and protein expression, leading to a significant increase in the percentage of PD-L1 positive breast cancer cells (11, 12). The above studies suggest that combining chemotherapy with immunotherapy can enhance the efficacy of immunotherapy.

Vascular endothelial growth factor (VEGF) is overexpressed in the vast majority of solid tumors and is widely recognized as a crucial factor mediating tumor angiogenesis (13, 14). Therefore, the blockade of VEGF/vascular endothelial growth factor receptor (VEGFR) signaling transduction has been acknowledged as a broad-spectrum anti-tumor therapeutic mechanism (15). Bevacizumab is a recombinant human immunoglobulin G (IgG) monoclonal antibody that targets VEGF to inhibit the formation of the VEGF and VEGFR-2 complex (16). It is currently widely used in the treatment of various solid tumors, including metastatic non-squamous non-small cell lung cancer (NSCLC) (17), metastatic renal cell carcinoma (18), epithelial ovarian cancer (19), breast cancer (20, 21), and malignant glioma (22).

More importantly, there is a strong correlation between VEGF and PD-1 expression in the tumor microenvironment (23). Studies have shown that anti-VEGF therapy can relieve the inhibition of dendritic cell (DC) maturation by VEGF-A, thereby promoting antigen presentation (24). It is well known that the downregulation of endothelial cell adhesion molecules and the expression of Factor-Related Apoptosis Ligand (FASL) are the main reasons for the reduced infiltration of T cells into tumor cells (25, 26). However, anti-VEGF therapy can reduce or eliminate the decrease in tumor T cell infiltration caused by the abnormal tumor vasculature mediated by VEGF-A, thereby promoting T cell infiltration into the tumor (27). Furthermore, anti-VEGF therapy can reverse the VEGF-induced expansion of myeloid-derived suppressor cells (MDSCs) and regulatory T cells (Tregs), relieve the immunosuppressive tumor microenvironment, and remodel the tumor microenvironment to increase immune infiltration (28). Blocking immune checkpoints can induce Th1 cells to secrete IFN-γ, promoting vascular normalization. Normalized blood vessels can further promote T cell activation, leading to more tumor vascular remodeling, thereby triggering a “positive feedback loop” of anti-angiogenic effects and ultimately producing long-term tumor control (29). Therefore, using a bispecific antibody to simultaneously block PD-1 and VEGF can provide a multi-targeted agent in the tumor microenvironment, exerting a synergistic effect (30). Based on the results of the IMbrave150 study (31), Atezolizumab (an anti-PD-L1 monoclonal antibody) combined with Bevacizumab was approved by the FDA in 2020 as a first-line treatment for advanced hepatocellular carcinoma. Additionally, based on the IMpower150 study results (32), the addition of Atezolizumab and Bevacizumab to first-line paclitaxel + carboplatin chemotherapy demonstrated PFS and OS benefits in patients with metastatic non-squamous NSCLC compared to chemotherapy plus Bevacizumab. Clinical benefits were also observed in patients with EGFR mutations or ALK-positive (EGFR-mut/ALK+) tumors after TKI resistance. Notably, a recent Phase Ib study evaluated the efficacy and predictive biomarkers of the PD-L1 inhibitor Benmelstobart (TQB2450) combined with the multi-target anti-angiogenic inhibitor Anlotinib in previously treated advanced TNBC patients. The study found that blood tumor mutational burden (bTMB) and maximum somatic allele frequency (MSAF) could predict patients’ ORR and PFS to the combined immune and anti-angiogenic therapy. Patients with both low bTMB and low MSAF achieved an ORR of 70% and a median PFS of 11.0 months, significantly outperforming other patient subgroups (33). All the above findings suggest a synergistic anti-tumor effect when PD-1/PD-L1 inhibitors are combined with anti-VEGF agents.

At present, chemotherapy combined with immunotherapy has become the standard first-line treatment for unresectable locally advanced or metastatic TNBC. IMpassion130 (34) is an international, multicenter, double-blind, placebo-controlled, randomized Phase III clinical study designed to evaluate the efficacy and safety of the PD-L1 inhibitor Atezolizumab combined with chemotherapy in treating unresectable locally advanced or metastatic TNBC, pioneering the use of immunotherapy in advanced TNBC. This clinical study enrolled a total of 902 patients, randomly assigned in a 1:1 ratio to the Atezolizumab plus nab-paclitaxel group and the placebo plus nab-paclitaxel group. The results showed that in the intention-to-treat (ITT) population, the PFS for the experimental and control groups was 7.2 months and 5.5 months, respectively (HR: 0.80, 95% CI: 0.69-0.92, P = 0.0025). In the PD-L1(+) population, the PFS for the experimental and control groups was 7.5 months and 5.0 months, respectively (HR: 0.62, 95% CI: 0.49-0.78, P<0.001). The results for the other primary endpoint, OS, showed that the median OS in the ITT population for the experimental and control groups was 21.3 and 17.6 months, respectively (HR = 0.84, 95% CI: 0.69-1.02, P = 0.08), while in the PD-L1(+) population, the median OS was 25.0 and 15.5 months, respectively (HR = 0.62, 95% CI: 0.45-0.86), demonstrating a survival benefit of nearly 10.0 months for the experimental group. The ORR in the ITT population for the experimental and control groups was 56.0% and 45.9% (P = 0.002), respectively, and in the PD-L1(+) population, it was 58.9% and 42.6% (P = 0.002), respectively. These findings indicate that application of Atezolizumab combined with nab-paclitaxel significantly improves PFS in TNBC patients compared to nab-paclitaxel monotherapy, with more pronounced benefits observed in the PD-L1(+) population.

KEYNOTE-355 (35) is a randomized, double-blind, global multicenter Phase III clinical study aimed at evaluating the efficacy and safety of Pembrolizumab combined with chemotherapy as a first-line treatment for TNBC patients. The trial enrolled a total of 847 patients, randomly assigned in a 2:1 ratio to the Pembrolizumab plus chemotherapy group or the placebo plus chemotherapy group. The chemotherapy regimen was the investigator’s choice of nab-paclitaxel, paclitaxel, or gemcitabine/carboplatin. The results showed that for patients with a Combined Positive Score (CPS) ≥10, the median PFS for the Pembrolizumab group vs. the placebo group was 9.7 months vs. 5.6 months (HR = 0.65). However, for patients with CPS ≥1, the median PFS for the two groups was 7.6 months vs. 5.6 months, with no statistically significant difference.Notably, this OS survival benefit was statistically significant in the CPS ≥10 subgroup (23.0 vs. 16.1 months; HR, 0.73; 95% CI 0.55-0.95; P = 0.019), whereas the difference did not reach significance in the CPS ≥1 cohort (17.6 vs. 16.0 months; HR, 0.86; 95% CI 0.72-1.04; P = 0.11) (36). This clinical study data further demonstrates that metastatic TNBC patients with high PD-L1 expression derive significant clinical benefit from first-line Pembrolizumab combined with chemotherapy compared to chemotherapy alone. However, TNBC patients with PD-L1 CPS ≥10 account for only approximately 35% from Asian populations (37), meaning that the majority of patients still cannot benefit from combination immunotherapy.

TORCHLIGHT (38)is a randomized, double-blind, placebo-controlled, multicenter Phase III clinical study designed to evaluate the efficacy and safety of Toripalimab combined with nab-paclitaxel (nab-P) versus placebo combined with nab-P in the first-line treatment of Chinese patients with newly diagnosed stage IV or recurrent/metastatic TNBC. This study met its primary endpoint: in the PD-L1-positive population (CPS≥1), compared to chemotherapy alone, Toripalimab combined with nab-P significantly improved patients’ PFS. The median PFS assessed by the Blinded Independent Central Review (BICR) was 8.4 months vs. 5.6 months, respectively (HR = 0.65 [95% CI 0.47-0.91]). In both the PD-L1positive and ITT populations, a clear trend of OS benefit was observed in the Toripalimab plus nab-P group (in the PD-L1-positive population, the median OS for the two groups was 32.8 months vs. 19.5 months, HR = 0.62 [95% CI 0.41-0.91]; in the ITT population, the median OS was 33.1 months vs. 23.5 months, HR = 0.69 [95% CI 0.51-0.93]). This study represents the first Phase III trial in China to demonstrate clinical efficacy in immunotherapy for advanced TNBC, further extanding the clinical benefits to patients with PD-L1 CPS ≥1 and filling the existing data void for immunotherapy in the Chinese demographic.

In addition, pivotal clinical studies on chemotherapy combined with anti-angiogenic therapy have also been conducted in advanced/metastatic TNBC. E2100 (NCT00028990) (39) is a randomized, double-blind Phase III study aimed at evaluating the efficacy and safety of Bevacizumab plus chemotherapy as a first-line treatment for metastatic breast cancer. This trial enrolled 722 subjects, randomly assigned to Bevacizumab plus paclitaxel group or placebo plus paclitaxel group. The results showed that in TNBC subgroup, the median PFS for the Bevacizumab plus paclitaxel group versus the placebo plus paclitaxel group was 8.8 months vs. 4.6 months (HR = 0.53). This clinical study data indicates that metastatic TNBC patients can derive significant clinical benefit from Bevacizumab combined with chemotherapy. RIBBON-1 (NCT00262067) (40) is a randomized, double-blind Phase III study designed to evaluate the efficacy and safety of Bevacizumab combined with chemotherapy as a first-line treatment for metastatic breast cancer. The study enrolled 1,237 patients, randomly assigned in a 2:1 ratio to Bevacizumab plus chemotherapy group or placebo plus chemotherapy group, with chemotherapy agents being taxanes, anthracyclines, or capecitabine. The results showed that in the TNBC subgroup analysis, the PFS for Bevacizumab plus taxanes/anthracyclines group vs. placebo plus taxanes/anthracyclines group was 6.5 months vs. 6.2 months (HR = 0.78); the PFS for Bevacizumab plus capecitabine group vs. placebo plus capecitabine group was 6.1 months vs. 4.2 months (HR = 0.72). This study data suggests that metastatic TNBC patients may benefit from Bevacizumab combined with chemotherapy. RIBBON-2 (NCT00281697) (41) is a randomized, double-blind Phase III study aimed at evaluating the efficacy and safety of Bevacizumab combined with chemotherapy as a second-line treatment for metastatic HER2-negative breast cancer, with chemotherapy agents including capecitabine, taxanes (paclitaxel, nab-paclitaxel, docetaxel), gemcitabine, and vinorelbine. The results showed that in the TNBC subgroup analysis, the PFS for Bevacizumab plus chemotherapy group vs. placebo plus chemotherapy group was 6.0 months vs. 2.7 months (HR = 0.494), and the OS was 17.9 months vs. 12.6 months (HR = 0.624). This clinical study data indicates that metastatic TNBC patients can still benefit from Bevacizumab combined with chemotherapy in the second-line setting.

Notably, in previous clinical studies of chemotherapy combined with immunotherapy for TNBC, taxanes (especially nab-paclitaxel) were mostly selected as the optimal partners for combination immunotherapy, such as in the IMpassion130, KEYNOTE-355, and TORCHLIGHT studies. However, the NCT04303741 clinical study (42) demonstrated that the “triple-drug” regimen of Eribulin combined with Apatinib and Camrelizumab still achieved a median PFS of 8.1 months, an ORR of 37%, and a DCR of 87% in refractory advanced TNBC patients who had previously received a median of three lines of treatment. This clinical study data provides further robust evidence supporting the efficacy of combining chemotherapy with anti-angiogenic agents and immunotherapy in advanced TNBC. Furthermore, the above research indicates that besides taxanes, Eribulin is another potential chemotherapy partner in anti-angiogenic and/or immune combination therapies.

Eribulin is a halichondrin-class, non-taxane microtubule dynamics inhibitor with established antineoplastic activity (43, 44). It is a structurally modified synthetic analogue of halichondrin B, a natural product isolated from the marine sponge *Halichondria okadai (*45). Eribulin possesses a novel mechanism of action distinct from other microtubule-targeted drugs—it specifically inhibits the growth phase of microtubules without affecting the shortening phase, binding to high-affinity sites at the growing ends of microtubules and leading to the sequestration of tubulin into non-functional aggregates (46). The landmark global Phase III EMBRACE study (47) showed that compared to the treatment of physician’s choice, Eribulin significantly prolonged the OS of patients with refractory metastatic breast cancer who had previously received multiple lines of treatment (13.1 months vs. 10.6 months), with an HR of 0.81, reducing the risk of death by 19%. Another randomized Phase III study (Study 301) (48) comparing Eribulin head-to-head with capecitabine in locally advanced or metastatic breast cancer previously treated with anthracyclines and taxanes showed that in the TNBC subgroup, Eribulin prolonged patient OS compared to capecitabine (14.4 months vs. 9.4 months), reducing the risk of death by 29.8% (p=0.006), further demonstrating the efficacy of Eribulin in advanced TNBC. More importantly, the EMBRACE and 301 studies consistently found that although Eribulin-induced peripheral neuropathy is common, its severity (Grade 3/4) is lower than that of paclitaxel and is usually reversible. Moreover, it remains active against cells that have developed paclitaxel resistance due to β-tubulin mutations. Therefore, Eribulin may still have good therapeutic effects on tumor cells resistant to other microtubule-targeted drugs. In addition, Eribulin can act as a tumor microenvironment modulator, exerting synergistic effects in improving vascular remodeling, reversing epithelial-mesenchymal transition (EMT), and ameliorating the immune microenvironment (promoting immune cell infiltration; decreasing FOXP3 expression, and increasing CD8+ T cell expression) (49, 50).

Ivonescimab is a novel bispecific antibody targeting PD-1/VEGF independently developed in China. Relying on its unique “tumor immunity + anti-angiogenesis” dual-target synergistic anti-tumor effect, it can both block the binding of PD-1 to PD-L1/L2 and inhibit the interaction between VEGF and its receptors. It has currently achieved breakthroughs in the treatment of multiple solid tumors (51, 52). The HARMONi-A (AK112-301) study is a randomized, double-blind, placebo-controlled, multicenter Phase III study (53, 54) aimed at evaluating the efficacy and safety of Ivonescimab combined with chemotherapy versus placebo combined with chemotherapy in patients with advanced non-squamous NSCLC who have failed prior EGFR-TKI treatment. The results showed that the PFS for Ivonescimab combined with chemotherapy versus placebo combined with chemotherapy was 7.1 months vs. 4.8 months (HR = 0.46), reducing the risk of disease progression or death by 54%. This represents a further improvement compared to the 7.2-month PFS of Sintilimab + bevacizumab + chemotherapy based on the ORIENT-31 study (55). Based on these excellent clinical study data, on May 24, 2024, Ivonescimab received its first approval from the National Medical Products Administration (NMPA) to be used in combination with chemotherapy (pemetrexed + carboplatin) for locally advanced or metastatic non-squamous NSCLC with EGFR mutations that has progressed after TKI therapy, making it the world’s first bispecific antibody drug with clear benefits after EGFR-TKI treatment progression. Furthermore, in an open-label, multicenter Phase II trial (NCT05227664), first-line treatment with Ivonescimab combined with paclitaxel or nab-paclitaxel for TNBC demonstrated remarkable efficacy, yielding an ORR of 80%, a disease control rate (DCR) of 100%, and a median PFS of 15.2 months (56).It is worth mentioning that Ivonescimab combined with chemotherapy also achieved significant efficacy in patients with low PD-L1 expression (CPS< 10), with the ORR of 79.3%, the median DoR was 9.9 months and the median PFS was 13.0 months. This exciting research result lays a solid foundation for conducting in-depth studies of Ivonescimab in TNBC and broadening the beneficiary population.

This study is a multicenter, open-label, single-arm clinical trial planning to enroll 29 patients with locally advanced or metastatic TNBC who have not previously received systemic anti-tumor therapy, are unsuitable for radical surgical resection or local therapy, or have experienced disease progression after radical surgical resection or local therapy. The study aims to evaluate the efficacy and safety of Eribulin combined with Ivonescimab as a first-line treatment for metastatic TNBC, providing new evidence-based medical support for the optimal first-line treatment of TNBC patients.

## Methods/design

### Objective

The study aims to evaluate the efficacy and safety of Eribulin combined with Ivonescimab as the first-line treatment for patients with locally advanced or metastatic TNBC.

### Trial design

This is a multicenter, open-label, single-arm interventional study. It plans to enroll patients with locally advanced or metastatic TNBC who have not received prior systemic anti-tumor therapy, are unsuitable for radical surgical resection or local therapy, or have experienced disease progression after radical surgical resection or local therapy. The study aims to evaluate the efficacy and safety of first-line treatment with Eribulin combined with Ivonescimab. According to RECIST v1.1 criteria, patients assessed as having CR (complete response), PR (partial response), or SD (stable disease) may continue treatment. For patients assessed as having PD (progressive disease), if the investigator determines that the patient may still derive clinical benefit from continued treatment after the first documented disease progression per RECIST v1.1, the patient may continue to receive study treatment until the occurrence of unacceptable toxicity or the investigator determines that there is no further clinical benefit, whichever occurs first, at which point treatment will be discontinued. The detailed study workflow is illustrated in **Figure 1**.

**Figure 1:**
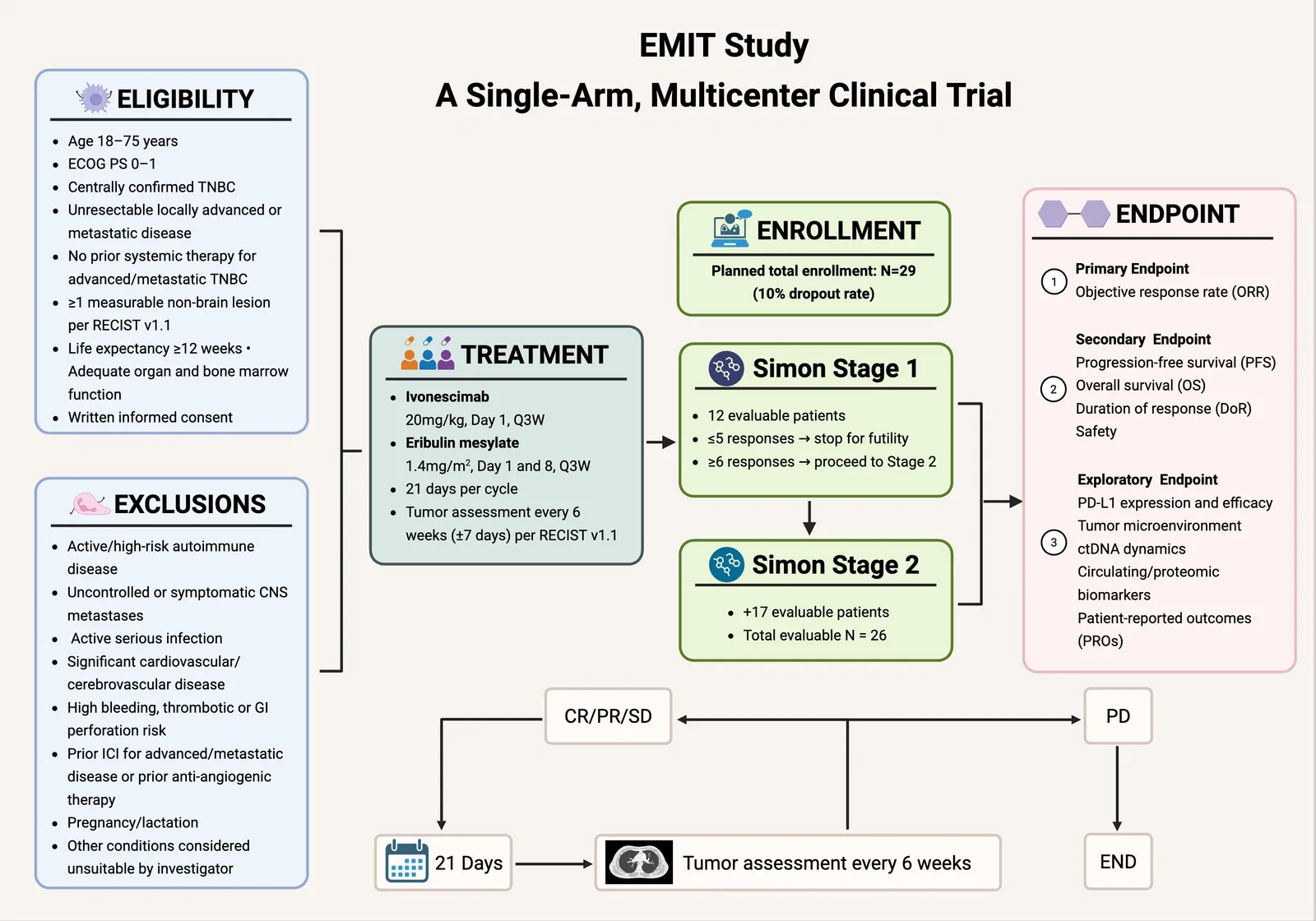
Detailed study workflow.

### Ethical standards and registration

This trial will be conducted in strict accordance with the ethical principles set forth in the Declaration of Helsinki. The study protocol has undergone formal review and was approved by the Ethics Committee of Sir Run Run Shaw Hospital, Zhejiang University School of Medicine on September 25, 2025 (Approval Number:2025-2772-01). In addition, this study has been prospectively registered with the Chinese Clinical Trial Registry (website: https://www.chictr.org.cn/) under the registration number ChiCTR2600117652.

### Informed consent

All eligible patients invited to participate will receive comprehensive information on the study, including potential benefits, foreseeable risks, and alternative treatments. Participation is entirely voluntary and based on informed consent. Participants may withdraw at any time without providing a reason and without any impact on their ongoing clinical care. All personal and clinical data will be handled in strict compliance with data protection regulations and kept confidential, accessible only to authorized study personnel. Prior to enrollment, written informed consent must be obtained after a thorough explanation of the study by a trained research team member.

### Study procedure

Patients who met the inclusion criteria received treatment with Eribulin combined with Ivonescimab: Ivonescimab was administered by intravenous infusion, at a recommended dose of 20mg/kg, once every 3 weeks; Eribulin was administered intravenously at a dose of 1.4mg/m^2^, within 2–5 minutes, with each 21-day period as a treatment cycle. The first dose was given on day 1 and the second dose on day 8 of each cycle. The treatment period: Tumor assessment was conducted every 6 weeks (± 7 days). Patients with CR, PR or SD could continue the treatment. For patients with PD, if the investigator, based on the RECIST v1.1 criteria, determined that the subject could continue to benefit from the study treatment after the first disease progression, the subject could continue to receive the study treatment until an intolerable toxicity occurred or the investigator determined that there was no longer a clinical benefit, whichever occurred first, to terminate the treatment.

### Inclusion criteria and exclusion criteria

The detailed inclusion and exclusion criteria for study participation are shown in **Table 1**.

**Table 1:** Inclusion criteria and exclusion criteria.

Inclusion criteria	Exclusion criteria
1. Age 18–75 years (inclusive), any sex. 2.ECOG PS 0–1. 3.Provide archived (within 2 years) and/or fresh tumor tissue; centrally confirmed TNBC (ER, PR, and HER2 negative): a) ER/PR negative: IHC<1% for both. b) HER2 negative: IHC 0 or 1+; if 2+, ISH/FISH must be negative. d) If molecular subtype differs between metastatic and surgical pathology, the latest metastatic pathology determines eligibility. 4.No prior systemic therapy for locally advanced, recurrent, or metastatic TNBC, and unsuitable for radical surgery/local therapy, or progressed after such treatments: a) Patients who relapsed after (neo)adjuvant chemotherapy (taxanes and/or anthracyclines) were considered treatment-naïve for advanced disease and eligible for this first-line study, provided the interval from the last dose of chemotherapy to recurrence was ≥12 months, ≥6 months for other agents (e.g., capecitabine, PD-1/PD-L1 inhibitors). b) Surgery alone: interval from surgery to first documented recurrence/metastasis ≥6 months. 5.At least one measurable (non-brain) lesion per RECIST v1.1: non-lymph node longest diameter ≥10 mm, lymph node short axis ≥15 mm (lesions previously irradiated or locally treated must have documented progression post-treatment; if target lesion biopsied at screening, interval to first dose ≥2 weeks). 6.Life expectancy ≥12 weeks. 7.Adequate organ and bone marrow function within 1 week before enrollment (per local lab normal range): a) Hematology: WBC ≥3.0×10^9^/L, ANC ≥1.5×10^9^/L, PLT ≥90×10^9^/L, HGB ≥9.0 g/dL. b) Hepatic: TBiL ≤1.5×ULN (≤3×ULN for liver metastases or Gilbert’s); ALT/AST ≤2.5×ULN (≤5×ULN for liver metastases); ALB ≥30 g/L; ALP ≤5×ULN for liver/bone metastases. c) Renal: Scr ≤1.5×ULN, or CrCl (Cockcroft-Gault) ≥60 mL/min. d) Coagulation: INR and APTT ≤1.5×ULN (therapeutic range acceptable for anticoagulated patients). e) Urinalysis: protein<2+; if ≥2+, 24h urine protein<1 g. 8.Women of childbearing potential: negative urine or serum pregnancy test within 72 h before first dose (serum required if urine positive or inconclusive). 9.Patients (and partners) willing to use effective contraception from screening to 90 days after last dose (e.g., double-barrier, condoms, oral/injectable contraceptives, IUD). 10.Voluntary participation with signed ICF. If unable to read/sign due to incapacity, legal guardian may provide consent. If illiterate, a witness must be present.	1.Other malignancy within 5 years, except cured thyroid cancer, cervical carcinoma *in situ*, basal/squamous cell skin cancer. 2.Participation in another investigational drug/device trial within 28 days before first dose; or prior anti-tumor therapy (chemotherapy, radiotherapy, targeted therapy, immunotherapy, endocrine therapy) within 4 weeks or 5 half-lives (whichever shorter); palliative radiotherapy ≥2 weeks before first dose; Chinese herbal medicine or non-specific immunomodulators (e.g., IL, interferon, thymosin, TNF) within 2 weeks before first dose (IL-11 for thrombocytopenia excluded). 3.Prior treatment-related AEs (except alopecia, ≤Grade 2 peripheral neuropathy, endocrine disorders stable on hormone replacement, and toxicities deemed safe by investigator) not recovered to ≤Grade 1 (NCI-CTCAE v5.0). 4.Prior Grade 3–4 irAE or treatment discontinuation due to irAE (except Grade 3 endocrine abnormalities stable on hormone replacement). 5.Active or history of autoimmune disease with risk of recurrence (e.g., SLE, RA, IBD, autoimmune thyroid disease, MS, vasculitis, glomerulonephritis), or high risk (e.g., post-organ transplant requiring immunosuppression). Exceptions: a) Type 1 diabetes stable on fixed insulin (HbA1c ≤8.0%). b) Autoimmune hypothyroidism on hormone replacement only. c) Skin conditions not requiring systemic therapy (e.g., eczema, rash<10% BSA, psoriasis without ocular symptoms). 6.Systemic corticosteroids (>10 mg/day prednisone or equivalent) or other immunosuppressants within 14 days before first dose. Inhaled, ophthalmic, topical, or ≤10 mg/day prednisone equivalent allowed (including premedication for contrast allergy). 7.Uncontrolled or symptomatic active CNS metastases. Asymptomatic brain metastases allowed if: ① stable on imaging and neurological exam for ≥4 weeks after treatment; ② if no imaging, stable on neurological exam for ≥4 weeks on corticosteroids, with prednisone ≤10 mg/day or equivalent at first dose. 8.Brainstem, leptomeningeal, spinal cord metastasis or compression. 9.Tumor invading or compressing vital organs (aorta, heart/pericardium, SVC, trachea, esophagus) or risk of esophagotracheal/pleural fistula; mediastinal lymph node metastasis invading trachea/main bronchus. 10.Active pulmonary tuberculosis or suspected active TB; infectious pneumonia requiring treatment. 11.History of ILD or non-infectious pneumonitis. 12.Any of the following infections: a) Active infection requiring systemic therapy within 2 weeks before screening. b) HIV antibody positive. c) Active hepatitis B or C. Inactive HBV carriers (HBV DNA<2000 IU/mL) or cured HCV (HCV RNA negative) allowed (if HBV DNA positive, antiviral therapy required ≥14 days before first dose; if HBsAg and/or HBcAb positive, investigator to assess need for antivirals). 13.Clinically significant cardiovascular/ cerebrovascular disease: a) CHF (NYHA III–IV). b) Severe/unstable angina or new-onset angina within 3 months. c) Acute MI within 6 months before screening. d) Severe arrhythmia requiring treatment (e.g., AF, VT, complete LBBB, complete block, TdP, familial/genetic arrhythmia, long QT syndrome). e) LVEF<50% on echocardiography. f) QTcF prolongation: male >450 ms, female >470 ms. g) Acute cerebrovascular event (e.g., cerebral infarction, hemorrhage, TIA, CVA, hypertensive crisis/encephalopathy) within 6 months before screening. h) Uncontrolled hypertension (resting SBP >160 mmHg or DBP >100 mmHg) despite antihypertensives. i) Other cardiac impairment affecting safety evaluation (e.g., myocardial ischemia, cardiomyopathy). 14.Untreated or incompletely treated conditions with high bleeding risk within 6 months before screening: a) Active gastric ulcer, GI bleeding, coagulopathy; imaging showing tumor invading major vessels, or judged likely to cause fatal hemorrhage. b) Arterial thrombosis/embolism or significant vascular disease (e.g., aortic aneurysm requiring repair, aortic dissection); DVT within 3 months before screening. c) Conditions predisposing to GI bleeding or perforation (e.g., duodenal ulcer, bowel obstruction, Crohn’s, UC, extensive gastrectomy/enterectomy); history of intestinal perforation/fistula not healed post-surgery; esophageal/gastric varices. 15.Clinically symptomatic, uncontrolled, or requiring repeated drainage of pleural, pericardial, or peritoneal effusion. 16.Uncontrolled comorbidities, including decompensated cirrhosis, nephrotic syndrome. 17.Prior exposure to immune checkpoint inhibitors(e.g. anti-PD-1, anti-PD-L1, anti-CTLA-4) for advanced or metastatic disease (prior neoadjuvant/adjuvant ICI therapy is permitted if the recurrence-free interval is >6 months, as per Inclusion Criterion 4a) or anti-angiogenic agents (e.g., bevacizumab, anlotinib, apatinib, lenvatinib, sorafenib, sunitinib, regorafenib, fruquintinib). 18.Prior stem cell, bone marrow, or solid organ transplant. 19.Surgery (except biopsy) within 4 weeks before screening, or surgical incision not fully healed. 20.Blood transfusion, G-CSF, GM-CSF, TPO, or EPO within 2 weeks before first dose. 21.Severe allergy history, or Grade 3–4 allergic reaction to other monoclonal antibodies. 22.Live virus vaccine within 30 days before screening or planned during study. 23.Psychiatric, psychological, familial genetic disease, or alcohol/drug addiction that may interfere with treatment, follow-up, compliance, or increase risk. 24.Pregnant or lactating women. 25.Local or systemic conditions not caused by malignancy, or tumor-related conditions with high medical risk or uncertain survival assessment (e.g., leukemoid reaction with WBC >20×10^9^/L, cachexia with >10% weight loss within 3 months before screening). 26.Unable to comply with study procedures or deemed unsuitable by investigator.

### Outcome measures

1. Primary Endpoint

Objective Response Rate (ORR): Proportion of evaluable patients whose best overall response is CR or PR per RECIST v1.1 from enrollment to disease progression.

2. Secondary Endpoints

2.1 Progression-Free Survival (PFS): Time from enrollment to first radiologically confirmed PD (RECIST v1.1) or death from any cause without documented progression.

2.2 Overall Survival (OS): Time from enrollment to death from any cause.

Duration of Response (DoR): Time from first documented CR or PR to PD or death (whichever occurs first), calculated only for patients with CR or PR as best response.

2.3 Safety: AEs (including SAEs) and laboratory abnormalities summarized by severity. The safety population includes all patients who received at least one dose of Eribulin plus Ivonescimab and have post-dose safety records.

3. Exploratory Biomarker Studies

3.1 PD-L1 Expression and Efficacy Correlation:

IHC assessment of PD-L1 (CPS) in tumor tissue; analysis of ORR and PFS across different expression levels to evaluate the exploratory association between baseline PD-L1 expression and clinical outcomes.

3.2 Tumor Microenvironment (TME) Characterization:

Multiplex IHC (mIHC) or RNA-seq to evaluate baseline TIL density and VEGF receptor expression, exploring efficacy differences between “hot” and “cold” tumors. Single-cell sequencing to analyze infiltration ratios of NK cells and CD8+ T cells.

3.3 Peripheral Blood Dynamic Monitoring:

3.3.1 ctDNA dynamics: NGS-based ctDNA abundance changes at baseline and during treatment; assess whether early ctDNA decline (molecular response) predicts clinical response earlier than imaging.

3.3.2 Angiogenesis-related factors: Serial measurement of VEGF, sVEGFR-2, etc., to evaluate target coverage by Ivonescimab and its correlation with efficacy.

3.3.3 Olink-based plasma proteomics: Plasma samples collected at baseline (C1D1), at every two cycles (e.g. C3D1, C5D1……) during tumor assessment and at end of treatment (EOT) (progression or toxicity-related discontinuation). Specifically, it will analyze immune activation markers (CXCL9, CXCL10, IFN-γ, IL-12), angiogenesis/remodeling markers (VEGF-A, VEGF-D, PLGF, sVEGFR-2, Ang-2), and endothelial-to-mesenchymal transition-related proteins. Furthermore, it will evaluate immunosuppressive factors (e.g., IL-6, IL-10, TGF-β), tumor microenvironment (TME) modulators, and potential resistance-associated proteins.

Notes: 1. Categorization of Analyses:

1. Prespecified Core Analyses: We have defined analyses utilizing readily accessible samples (e.g., peripheral blood and archival tumor tissues) as core analyses. These include ctDNA analysis, plasma proteomics, and mIHC. These are mandatory for all enrolled patients to ensure a robust baseline and longitudinal dataset.
2. Optional/Sample-Availability-Dependent Analyses: Analyses requiring fresh tumor biopsies with high RNA quality, such as RNA-seq and single-cell sequencing (scRNA-seq), have been designated as optional. These will be performed only when patients consent to optional biopsies and the obtained tissue meets the stringent quality control criteria.

2. Sampling Time Points:

1. Blood samples (ctDNA & proteomics): Collected at baseline, at every two cycles (e.g. C3D1, C5D1……) during tumor assessment, and at EOT.
2. Tumor tissues (mIHC, RNA-seq, scRNA-seq): Archival or fresh biopsies collected at baseline; optional fresh biopsies collected at EOT.

4.Patient-Reported Outcome (PRO) Assessment (Optional)

EORTC QLQ-C30 or QLQ-BR23 questionnaires to evaluate symptom burden and quality of life during treatment, particularly changes in physical and social function after Eribulin plus Ivonescimab, emphasizing dual benefit of survival time and quality of life.

### Safety evaluation

All adverse events (AEs) occurring during the study will be recorded and assessed. For each reported AE, the following details will be documented in the CRF: (1) date and time of onset; (2) severity (per CTCAE v5.0: Grade 1 = mild, Grade 2 = moderate, Grade 3 = severe, Grade 4 = life-threatening, Grade 5 = death); (3) relationship to study drug (definitely related, probably related, possibly related, definitely unrelated, unassessable); (4) medical intervention taken; (5) clinical outcome (resolved, resolving, persisting, or fatal); (6) duration of follow-up and results; and (7) any decision regarding modification of intervention or participant withdrawal from the trial.

### Quality control

All investigators must complete professional training prior to study initiation to ensure standardized management. The training covers subject assessment, strict adherence to study inclusion/exclusion criteria, familiarity with the study protocol, and CRF completion guidelines (including accurate recording of results, adverse events, concomitant medications, and compliance data). All study data will be recorded in CRFs and entered into the electronic data capture system by independent research personnel. Subject dropout will be tracked throughout the study. This trial is supervised by the Clinical Research Center of Sir Run Run Shaw Hospital, Zhejiang University School of Medicine. The supervisory team is responsible for data review, verifying consistency between source data and recorded data, and ensuring the accuracy and quality of study data throughout the trial.

### Statistical methods

#### Sample size

The primary endpoint is ORR. Assuming ORR improves from 50% (based on IMpassion 130 and TORCHLIGHT data) to 75% (56), sample size is calculated using Simon’s two-stage optimal design, with a 10% dropout rate.

1. Statistical Parameters

P0 (null hypothesis ORR): 50% (0.50), P1 (alternative hypothesis ORR): 75% (0.75), α (Type I error): 0.05, β (Type II error): 0.20, power = 1− β = 80%, Dropout rate: 10%.

2. Simon’s Two-Stage Optimal Design

This design minimizes the expected sample size under the null hypothesis (ineffective drug), allowing early termination if the regimen is futile.

Stage 1: Enroll n1 = 10 patients. If ≤ 5 responses are observed, the trial is terminated early for futility. If ≥ 6 responses are observed, the trial proceeds to the second stage.

Stage 2: Enroll an additional n2 = 16 patients, bringing the total sample size to N = 26 patients.

Final decision: Among the total 26 patients, if ≤16 responses are observed (r=16), the drug is considered ineffective. If ≥17 responses are observed, the null hypothesis is rejected, and the drug is considered effective.

3. Adjusting for Dropout Rate

After calculating the actual evaluable sample size required for statistical purposes (N = 26), the total sample size to be enrolled (N_total) must be calculated based on the 10% dropout rate:


N_total=N/(1−”Dropout rate”)=26/(1−0.10)=28.89

Rounding up, the final planned total enrollment is 29 patients.

4. Summary

Planned enrollment for Stage 1: 10/0.9 ≈12 patients (10 evaluable).

Planned enrollment for Stage 2: 16/0.9 ≈17 patients (16 evaluable).

Total planned enrollment: 29 patients.

### Statistical analysis

The sample size calculation was performed using the clinfun package in R software (R version 4.4.1 for Statistical Computing). All efficacy and safety analyses were performed on the analysis population, which included all enrolled patients who received at least one post-baseline tumor assessment.

Baseline characteristics, including demographics and disease characteristics of the subjects, will be summarized using descriptive statistics. Continuous variables will be presented as mean ± standard deviation (SD) or median (interquartile range, IQR) depending on their distribution; categorical variables will be presented as frequencies and percentages. The primary endpoint of the study is ORR. The ORR and its 95% CI will be calculated using the Clopper-Pearson exact method. Survival outcomes, such as PFS and OS, will be analyzed using the Kaplan-Meier method to calculate the median survival time and its 95% CI. Adverse events (AEs) and serious adverse events (SAEs) will be graded and described according to the CTCAE v5.0 criteria, and the incidence of each adverse reaction will be calculated.

## Discussion

TNBC is highly malignant and has an extremely poor prognosis due to the lack of effective therapeutic targets (57). In recent years, immune checkpoint inhibitors combined with chemotherapy (such as the IMpassion130 (34), KEYNOTE-355 (35), and TORCHLIGHT (38) studies) have established their standard status in the first-line treatment of advanced TNBC. However, existing regimens still face two major limitations. First, the significant survival benefits of combined chemoimmunotherapy are mainly restricted to the PD-L1 high-expression population (accounting for approximately 35% of TNBC patients), leaving the majority of patients unable to benefit. Second, previous regimens mostly rely on taxanes as the chemotherapy backbone. The severe toxic side effects, such as peripheral neuropathy (58, 59), and the tendency for drug resistance severely impair patients’ quality of life and long-term treatment compliance. Therefore, exploring novel combination strategies that can expand the population benefiting from immunotherapy and offer better tolerability is an urgent clinical need in the current TNBC field (6).

VEGF in the tumor microenvironment not only promotes angiogenesis but also mediates immunosuppression by inhibiting dendritic cell maturation and increasing immunosuppressive cells (such as MDSCs and Tregs). As a novel PD-1/VEGF bispecific antibody, Ivonescimab can simultaneously block immune checkpoints and angiogenesis pathways, triggering a “positive feedback loop” of vascular normalization and T cell activation. A recent phase II study published in Nature Medicine evaluating Ivonescimab in combination with chemotherapy as a first-line treatment for advanced TNBC, which achieved an 80% ORR and 100% DCR (disease control rate) (56). Most notably, in the PD-L1 low-expression (CPS< 10) population, this regimen still achieved an ORR of 79.3%. This fully demonstrates that the “immunity + anti-angiogenesis” dual-target synergistic mechanism can effectively remodel the immune microenvironment, potentially breaking the dependence of traditional PD-1/PD-L1 monoclonal antibodies on high PD-L1 expression and significantly broadening the beneficiary population of TNBC.

In the search for alternatives to taxanes, Eribulin has shown highly promising advantages. As a non-taxane microtubule dynamics inhibitor, Eribulin not only maintains high activity against taxane-resistant tumor cells but also has a lower incidence of Grade 3/4 neurotoxicity, which is usually reversible (as shown in the EMBRACE (47) and Study 301 trials (60)). More importantly, Eribulin possesses unique tumor microenvironment-modulating functions, capable of reversing EMT, promoting tumor vascular remodeling, and increasing CD8+ T cell infiltration. The “triple-drug” regimen (Eribulin + Apatinib + Camrelizumab) led by Academician Erwei Song (42) achieved a median PFS of 8.1 months and an ORR of 37% in heavily pretreated refractory TNBC, providing solid evidence-based support for the strong synergistic anti-tumor effect of Eribulin combined with anti-angiogenic and immunotherapies.

In summary, this study innovatively proposes the use of Eribulin combined with Ivonescimab as a first-line treatment for locally advanced or metastatic TNBC. This powerful combination of “microenvironment-modulating chemotherapy + PD-1/VEGF bispecific antibody” aims to maximize the anti-tumor immune response through multi-pathway synergistic mechanisms. We expect that this regimen will not only further improve the ORR and PFS of TNBC patients and demonstrate preliminary clinical activity regardless of baseline PD-L1 expression levels but also effectively avoid the severe toxicities associated with traditional multi-line chemotherapy or taxanes, thereby improving patients’ quality of life. The implementation of this study will provide novel evidence-based medical support for the first-line treatment of advanced TNBC and is expected to bring superior survival benefits to this patient population with an extremely poor prognosis.

## Limitations

Several limitations of this study should be acknowledged. First, the single-arm, non-randomized design precludes a direct comparison with the current standard of care (e.g., chemotherapy plus pembrolizumab). Consequently, it is challenging to definitively isolate the true treatment effect of the combination regimen from potential confounding factors, such as patient selection bias (e.g., enrolling patients with more favorable performance status or lower tumor burden) or the natural course of the disease. Second, the open-label nature of the trial introduces the potential for investigator assessment bias, particularly when evaluating borderline lesions or reporting adverse events. As this was designed as an exploratory, proof-of-concept study, a BICR was not mandated. To mitigate this limitation, all tumor assessments strictly adhered to RECIST v1.1 criteria, and radiological images have been prospectively archived to facilitate retrospective independent audits if necessary. Third, the relatively small sample size restricts the statistical power for robust subgroup analyses. Given the substantial heterogeneity of TNBC, evaluating efficacy differences across key clinical subgroups—such as PD-L1 expression, germline BRCA mutation status, and specific metastatic sites (e.g., liver or lung or bone)—is of great clinical interest; however, our findings in these subgroups remain exploratory and require cautious interpretation.

Despite these limitations, this study provides crucial preliminary evidence for an innovative triplet regimen of “chemotherapy + immunotherapy + anti-angiogenesis” in the first-line setting. As a novel microtubule inhibitor, Eribulin not only exerts direct cytotoxicity but also remodels the tumor microenvironment to promote vascular normalization and immune cell infiltration. Concurrently, Ivonescimab dually blocks the critical pathways of immunosuppression and angiogenesis. The combination of these agents demonstrates a promising synergistic strategy of “chemotherapy-promoted immunity and bispecific antibody-enhanced efficacy.” While these early efficacy signals cannot immediately alter clinical practice, they highlight a potential strategy to overcome immunotherapy resistance, strongly supporting further evaluation of this regimen in larger, randomized controlled trials for patients with advanced TNBC.

## Conclusions

This study aims to evaluate the efficacy (primary endpoint: ORR; secondary endpoints: PFS, OS, DoR) and safety of Eribulin combined with Ivonescimab as first-line treatment for unresectable locally advanced or metastatic TNBC, and to explore potential predictive biomarkers associated with efficacy as well as the correlation between PD-L1 expression and efficacy. This study is not only a supplement to the existing chemo-immunotherapy combination paradigm, but also probably provides an optimal precision first-line treatment option for patients with low PD-L1 expression, holding significant scientific significance and socio-economic value.
